# Meta‐analysis of Alzheimer’s disease clinical trial time savings results

**DOI:** 10.1002/alz.093562

**Published:** 2025-01-09

**Authors:** Suzanne B. Hendrix, Kent Hendrix, Benjamin A Haaland, Patrick P O'Keefe, Newman Knowlton, Craig Mallinckrodt, Samuel P. Dickson

**Affiliations:** ^1^ Pentara Corporation, Salt Lake City, UT USA

## Abstract

**Background:**

Alzheimer’s disease (AD) clinical trials led to the recent successes with monoclonal antibodies targeting amyloid, which opens up many new directions for research into treatment for AD in the future. Gaining greater understanding from these successes and failures will help researchers to focus their efforts on avenues that have the highest potential benefit.

**Method:**

We performed a meta‐analysis of over a hundred studies of 70+ AD treatments. A global statistical test (GST) combining ADAS‐cog, ADCS‐ADL and CDR‐sb was used to assess the overall efficacy in these studies, in a fair way across multiple outcomes. Disease modifying treatment effects and symptomatic effects impacting all disease symptoms will perform better on this GST than treatments impacting only one symptom. In addition, a false positive effect is less likely to occur on all 3 outcomes simultaneously, making the GST a more reliable outcome for detecting true treatment effects. Composite scores, ADCOMS and iADRS, were used to target true disease progression in the lecanemab and donanemab phase 2 studies, respectively, and have similar advantages to a GST. A similar approach would have possibly averted much of the controversy surrounding the aducanumab approval.

**Result:**

Some studies that were previously determined to be failures show some indication of positive effects. And conversely, some studies previously thought to be promising are shown to be more clearly failures. Meta‐analyses combining similar treatments across programs illuminate overlooked mechanisms as well as more conclusive failures.

**Conclusion:**

The success and failure of Alzheimer’s treatments is partly hidden by including 3 different domains of clinical efficacy: cognition, function and global. True treatment efficacy and true lack of efficacy are much easier to detect with multiple, combined outcomes.

